# Mitochondria-targeted antioxidants as highly effective antibiotics

**DOI:** 10.1038/s41598-017-00802-8

**Published:** 2017-05-03

**Authors:** Pavel A. Nazarov, Ilya A. Osterman, Artem V. Tokarchuk, Marina V. Karakozova, Galina A. Korshunova, Konstantin G. Lyamzaev, Maxim V. Skulachev, Elena A. Kotova, Vladimir P. Skulachev, Yuri N. Antonenko

**Affiliations:** 1 Belozersky Institute of Physico-Chemical Biology, Lomonosov Moscow State University, Moscow, 119991 Russia; 2 Department of Chemistry, Lomonosov Moscow State University, Moscow, 119991 Russia; 3 Skolkovo Institute of Science and Technology, Skolkovo, 143026 Russia; 4 Vavilov Institute of General Genetics, Russian Academy of Science, Moscow, 117971 Russia; 5 Institute of Mitoengineering, Lomonosov Moscow State University, Moscow, 119991 Russia

## Abstract

Mitochondria-targeted antioxidants are known to alleviate mitochondrial oxidative damage that is associated with a variety of diseases. Here, we showed that SkQ1, a decyltriphenyl phosphonium cation conjugated to a quinone moiety, exhibited strong antibacterial activity towards Gram-positive *Bacillus subtilis, Mycobacterium sp*. and *Staphylococcus aureus* and Gram-negative *Photobacterium phosphoreum* and *Rhodobacter sphaeroides* in submicromolar and micromolar concentrations. SkQ1 exhibited less antibiotic activity towards *Escherichia coli* due to the presence of the highly effective multidrug resistance pump AcrAB-TolC. *E. coli* mutants lacking AcrAB-TolC showed similar SkQ1 sensitivity, as *B. subtilis*. Lowering of the bacterial membrane potential by SkQ1 might be involved in the mechanism of its bactericidal action. No significant cytotoxic effect on mammalian cells was observed at bacteriotoxic concentrations of SkQ1. Therefore, SkQ1 may be effective in protection of the infected mammals by killing invading bacteria.

## Introduction

At present, antibiotics are indispensable to treat bacterial infections. Unfortunately, resistance of bacteria to conventional antibiotics is a fast growing problem, causing millions of deaths every year and demanding seeking new antibiotics. The situation today is getting more desperate due to emergence and spread of bacterial strains, which are resistant to the majority of currently available antibiotics. Therefore, there is an urgent need to propose strategies that can resolve the problems of growing bacterial resistance. One of promising approaches is to apply appropriate membrane-active agents. Of these, quaternary ammonium (QACs) and phosphonium (QPCs) compounds^1–9^ have been used as antiseptics and disinfectants since the 1930-ies. The attention to their antimicrobial properties has been elevated after discovering enhancement of their efficacy in polymeric materials^10, 11^. Among membrane-active systemic antimicrobials, daptomycin, a membrane-permeabilizing cyclic anionic lipopeptide^12^ and cationic peptide nanoparticles^13, 14^ have recently attracted special attention. Of note, a series of studies have reported the development of high-level resistance to daptomycin in several bacterial species^15, 16^.

Operation of energy-coupled efflux systems causing antimicrobial multidrug resistance (MDR) has nowadays become a subject of intensive studies due to the importance of MDR as a serious health-threatening problem. Drug efflux could be prevented by using uncouplers of oxidative phosphorylation, i.e. compounds capable of decreasing an electrochemical gradient of protons across a bacterial cytoplasmic membrane called proton motive force (PMF). By decreasing PMF, uncouplers would inhibit operation of proton-dependent multidrug efflux systems^17^, suppress ATP synthesis and impede numerous metabolic processes. Therefore, targeting the PMF can be a promising approach to combat bacterial infections^18–24^.

Mitochondria-targeted antioxidants (MTAs), a wide range of compounds having an antioxidant group linked to a mitochondria-targeted moiety, such as triphenylphosphonium (TPP^+^) cation, are exemplified by TPP^+^-conjugated ubiquinone (MitoQ)^25^, plastoquinone (SkQ1)^26^ (for structure, see Supplementary Fig. S1), tocopherol^27^, lipoic acid^28^, spin traps^29^, the peroxidase mimetic Ebselen^30^, etc. MTAs are widely used in experiments for evaluating the impact of mitochondria on different pathological processes involving oxidative stress^31^. The ability of MTAs to alleviate mitochondrial oxidative damage and thereby improve the outcome of the pathology has been assessed *in vivo* in a number of murine disease models following the oral or intraperitoneal administration of MitoQ and SkQ1^26^. Along with the antioxidant action, hydrophobic cations with delocalized charge, like SkQ1, were shown to exert protonophore-like activity in artificial and mitochondrial membranes in the presence of fatty acids^32^.

Despite extensive studies, there is a lack of knowledge on the antimicrobial action of SkQ1 and MitoQ. Quite recently, an inhibitory effect of alkyl triphenyl phosphonium cations (C_n_-TPP) and SkQ1 on the growth of the Gram-positive bacterium *B. subtilis* has been demonstrated^33^; the effect increased with growing lipophilicity of C_n_-TPP. The growth of the Gram-negative bacterium *E. coli* was insensitive to C_n_-TPP and SkQ1. Different toxic effects on Gram-positive and Gram-negative bacteria species were explained by different permeability of bacterial cell envelopes for C_n_-TPP and SkQ1.

In the present work, we studied the antimicrobial action of SkQ1 on several bacteria including three Gram-positive (*B. subtilis*, *Mycobacterium sp., S. aureus*) and three Gram-negative (*E. coli*, *P. phosphoreum, R. sphaeroides*) bacteria. It was found that SkQ1 suppressed bacterial growth at submicromolar to tens micromolar concentrations, depending on the particular bacteria. *E. coli* was the most resistant to SkQ1, the resistance being obviously linked to the activity of AcrAB-TolC pump system.

## Results

### AcrAB-TolC transporter is responsible for *E. coli* resistance to SkQ1

**Figure 1:**
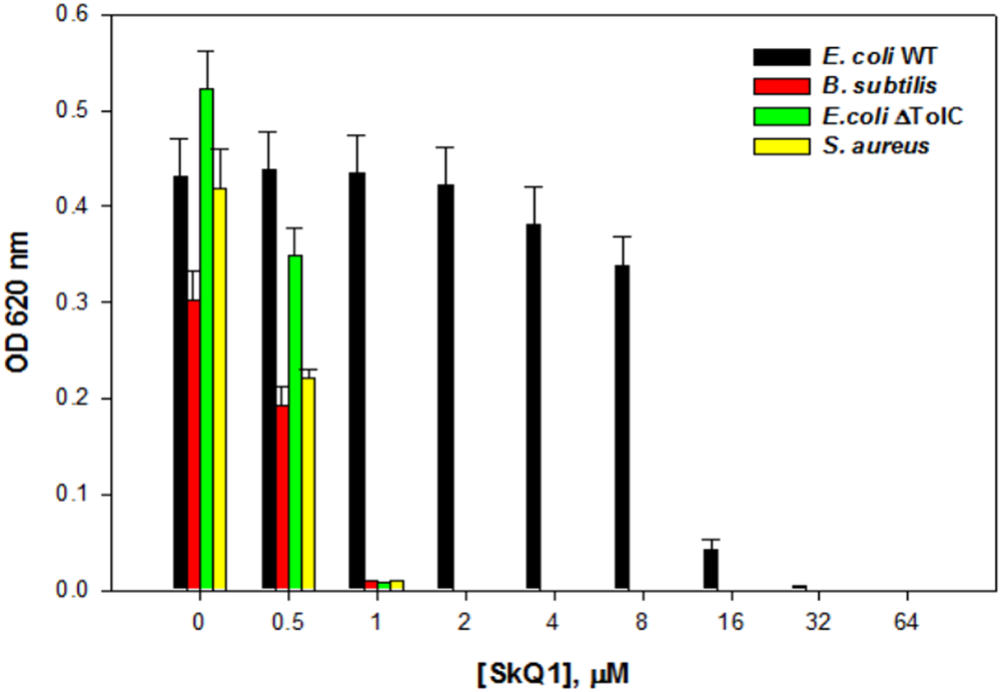
Dilution Antimicrobial Susceptibility Test. Bacterial growth was observed visually alongside the OD620 measurement.

To determine the difference in sensitivity to SkQ1 associated with cell envelope permeability, we selected two bacteria: Gram-positive *B. subtilis* and Gram-negative *E. coli*. We showed that micromolar concentrations of SkQ1 (Fig. 1 and Supplementary Fig. S2A) suppressed the growth of *B. subtilis*, while being ineffective in the case of *E. coli*. The effective concentration of SkQ1 for *E. coli* was tens of micromoles (Fig. 1 and Supplementary Fig. S2B). Lower concentrations of SkQ1 caused a delay in the growth kinetics. Importantly, SkQ1 acted as a bactericidal agent at concentrations higher than minimal inhibitory concentration (MIC), as evidenced by colony-forming units (CFU) counting (data not shown).

The relatively weak antibacterial action of SkQ1 towards *E. coli* could be due to an ability of this bacterium to expel a wide range of molecules out of cells by using efflux pumps. Actually, in Gram-negative bacteria, inner membrane transporters of the resistance-nodulation-cell division (RND) family, forming a tripartite complex with periplasmic adaptor proteins and an outer membrane channel protein, are effective in generating resistance to a plethora of agents. In *E. coli*, TolC, an outer membrane protein channel^34^, interacts with a variety of inner membrane transporters, thereby enabling the bacteria to expel structurally diverse molecules, such as enterotoxins^35^, antibiotics and antibacterial peptides, bile salts, and some other organic compounds.

Bearing in mind that TolC is a keystone component of several RND transporters in *E. coli* ^36^, we measured antibacterial action of SkQ1 on the TolC-deleted *E. coli* strain. The *ΔtolC E. coli* mutant demonstrated similar sensitivity to SkQ1 as *B. subtilis*: 1 µM of SkQ1 completely suppressed the growth of this mutant (Fig. 1 and Supplementary Fig. S2C). This result suggests that TolC-requiring transporters are responsible for resistance of *E. coli* cells to SkQ1. It should be noted that the *ΔtolC* mutant of *E. coli* demonstrated similar sensitivity to dodecyl triphenyl phosphonium (C_12_TPP), as to SkQ1 (data not shown), which is in agreement with the data obtained earlier on *B. subtilis* ^33^.

To identify transporter proteins requiring TolC for their functioning, we analyzed literature data on protein–protein interactions by using *STRING* database^37^. TolC functional partner prediction was performed for two *E. coli* K12 strains (W3110 and MG1655) at the highest confidence score (0.990). By combining the data for W3110 and MG1655, we revealed that AcrB, AcrD, AcrEF, MdtABC, MdtEF, MacAB, and EmrAB required TolC. Based on these data, we selected the TolC-required transporters panel for deletion analysis.

**Figure 2:**
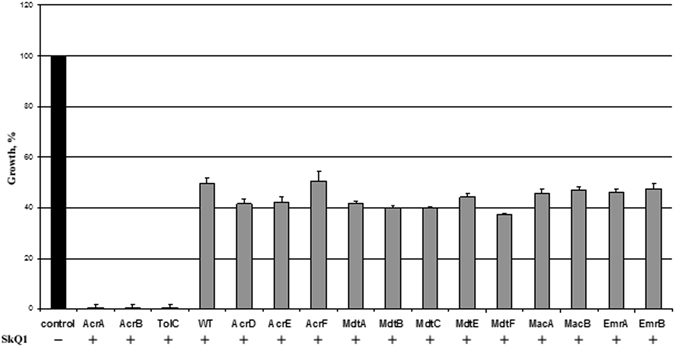
Growth of *E. coli* strains having deletions in various transporters in the presence of 10 μM SkQ1. SkQ1 was added at “0” time to the LB medium. Growth was evaluated after 15–35 h incubation at 30 °C or 37 °C by absorbance at 620 nm. The growth of WT *E. coli* cells in the absence of SkQ1 is referred as a control. The data points represent mean ± SD of three experiments.

Deletion of the transporter genes *acrD, acrE, acrF, mdtA, mdtB, mdtC, mdtE, mdtF, macA, macB, emrA, emrB* did not affect the sensitivity of *E. coli* cells to SkQ1 (Fig. 2, grey columns), therefore, all of them are not involved in the efflux of SkQ1 from wild type *E. coli* cells. By contrast, deletion of *acrA* and *acrB* genes dramatically increased the sensitivity of *E. coli* cells to SkQ1 (Fig. 2), hence, the AcrAB pump is the only pump responsible for SkQ1 resistance of *E. coli* cells. These data suggested that the AcrAB-TolC transporter is competent in expelling SkQ1 from *E. coli* cells.

It is known that TPP^+^ is expelled from *E. coli* cells by EmrE, a small multidrug resistance transporter^38^. Unfortunately, we were unable to detect any activity of the EmrE pump by using an EmrE deletion mutant (data not shown). We cannot exclude that this feature is related to the structural difference between TPP^+^ and SkQ1. Moreover, EmrE activity towards SkQ1 could be masked by the pronounced AcrAB-TolC activity.

**Figure 3:**
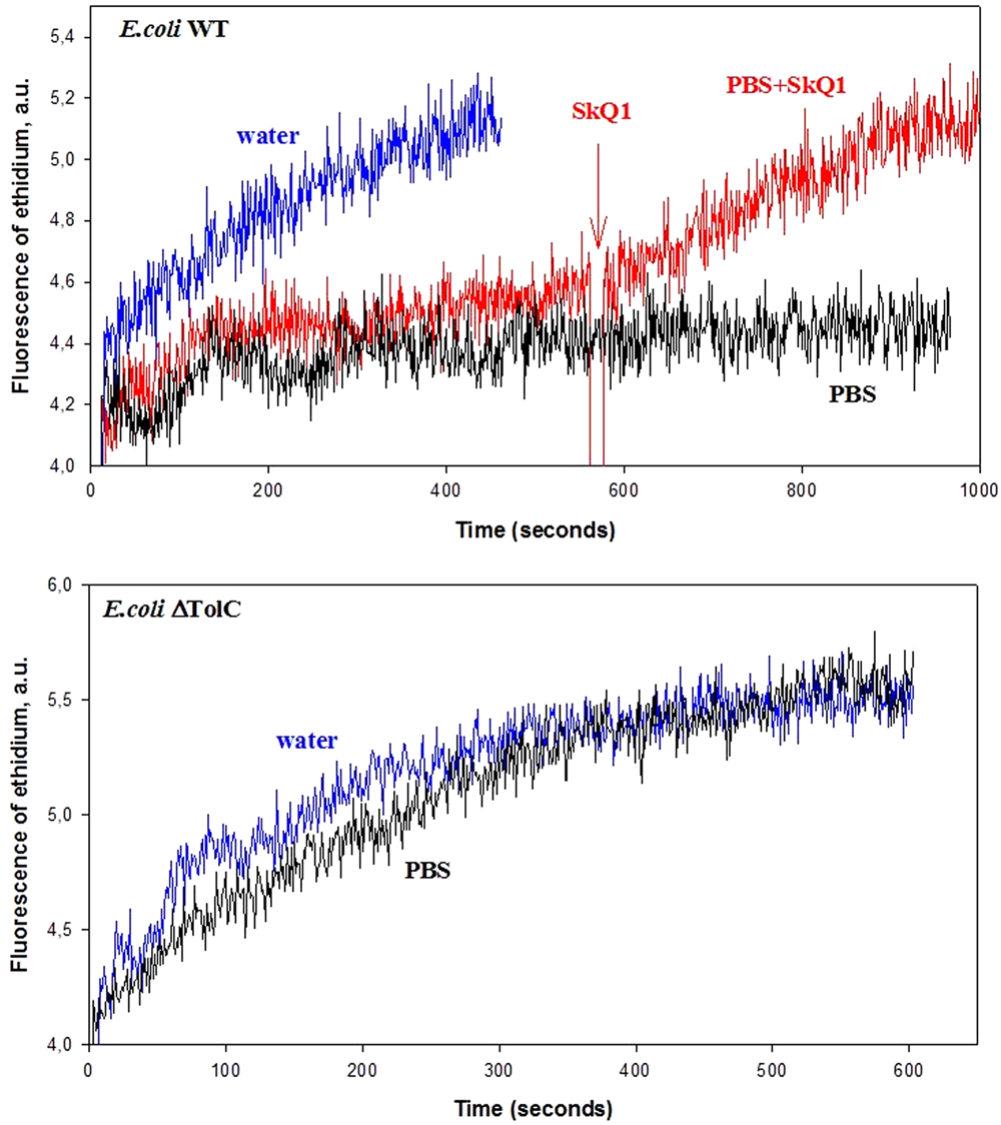
Effect of SkQ1 on ethidium bromide accumulation by WT (top panel) and Δ*tolC* (bottom panel) *E. coli* cells. *E. coli* cells were added to PBS with 20 μM ethidium bromide (black) and SkQ1 was added subsequently after 8 min of incubation (red). Osmotic shock by deionized water was used as a control (blue).

The conclusion that AcrAB-TolC is able to transport SkQ1 out of *E. coli* cells implies that SkQ1 could competitively inhibit efflux of other compounds mediated by AcrAB-TolC. To this end, we studied the effect of SkQ1 on the uptake of the cell membrane-permeant fluorescent dye ethidium bromide, known as a good substrate for the AcrAB-TolC system^39^. Ethidium is widely used in spectrofluorometric studies because of striking fluorescence enhancement it displays upon binding to nucleic acids. According to ref. 40, ethidium cation is unable to stain DNA of wild type *E. coli* cells up to 800 μg/ml due to the functioning of AcrAB-TolC pump, expelling ethidium out of cells^41^. Accumulation of ethidium inside cells can be measured by an increase in its fluorescence intensity^42^. Figure 3 shows the fluorescence of ethidium at a low concentration. The fluorescence was stable in time in the presence of *E. coli* cells (Fig. 3, top panel, black curve). The addition of SkQ1 induced an increase in the ethidium fluorescence (apparently, due to the cellular uptake of ethidium; Fig. 3, top panel, red curve), comparable to that caused by osmotic shock (Fig. 3, top panel, blue curve). Thus, it can be concluded that SkQ1 inhibited the efflux of ethidium either as a competing substrate of the AcrAB-TolC pump or as the uncoupler inactivating the pump by membrane deenergization via fatty acid protonophoric cycling (see below). With the *ΔtolC* mutant, the fluorescence of ethidium increased after the addition of the cells to isotonic solution, similar to the case of osmotic shock (Fig. 3, bottom panel), thereby confirming the involvement of the TolC protein in expelling ethidium out of WT *E. coli* cells.

**Figure 4:**
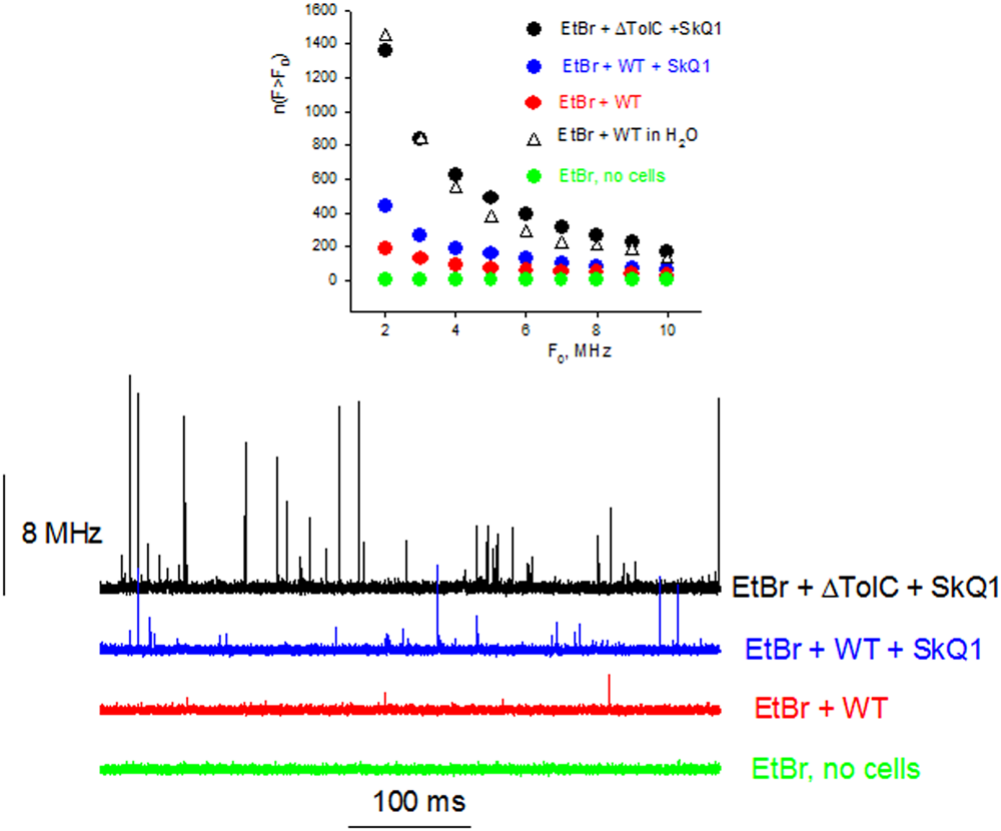
Effect of SkQ1 (10 µM) on the accumulation of ethidium bromide by *E. coli* WT and Δ*tolC* cells, monitored by FCS. Fluorescence intensity traces of ethidium (10 nM) were recorded with the FCS set-up in the presence or absence of bacterial cells (10^6^ per ml of PBS). Insert: Corresponding dependences of the number of peaks with the fluorescence intensity F exceeding the threshold F_0_, n(F > F_0_), on the value of F_0_.

To further confirm that AcrAB-TolC was a transporter for ethidium and SkQ1, we applied fluorescence correlation spectroscopy (FCS) for measuring ethidium accumulation in *E. coli* cells^43–45^. FCS measures fluctuations in the emission signal of a small number of fluorescent molecules diffusing into and out of the focus volume of an excitation laser. The ethidium solution without bacterial cells gave a fluorescence signal with low-amplitude fluctuations (Fig. 4, green curve), because there was a large number of free ethidium molecules in the confocal volume. In the case of incubation of *E. coli* cells in the presence of ethidium, the fluorescence recording contained peaks of rather high amplitude (Fig. 4, red curve), which corresponded to the appearance of cells bearing a large number of ethidium molecules. When WT *E. coli* cells were incubated with SkQ1 in the presence of ethidium, the high-amplitude peaks became more frequent (Fig. 4, blue curve), thus confirming the increase in the number of stained cells in the presence of SkQ1. The amplitude of the peaks was significantly larger, if *ΔtolC E. coli* cells were incubated with ethidium (Fig. 4, black curve). Measurements were conducted under the conditions of stirring the solution in order to improve statistics of signal recording. Inset in Fig. 4 presents plots of the number of peaks detected during 30 seconds versus the amplitude. Thus, the FCS method confirmed that SkQ1 competitively blocked the pump transporting ethidium.

Remarkably, in the absence of AcrA or AcrB proteins, the function of AcrAB-TolC transporter to expel SkQ1 was not substituted by another TolC-requiring pump, such as EmrAB-TolC, which is responsible for expelling other compounds, such as сarbonyl cyanide-*4*-(trifluoromethoxy)phenylhydrazone (FCCP), carbonyl cyanide *m*-chlorophenyl hydrazone (CCCP) and 2,4-dinitrophenol (DNP) from bacterial cells^46, 47^.

### Antibacterial activity of MTAs

To further test the idea that the cell envelope permeability for SkQ1 depends on the existence of transporters capable of expelling SkQ1 out of bacterial cells, we selected several additional Gram-negative (*P. phosphoreum* and *R. sphaeroides*) and Gram-positive bacteria *(S. aureus* and *Mycobacterium sp*.), differing in cell envelope composition. Our experiments showed that micromolar concentrations of SkQ1 suppressed the growth of *Mycobacterium sp*. (Supplementary Fig. S2D), *S. aureus* (Fig. 1 and Supplementary Fig. S2E), *P. phosphoreum* (Supplementary Fig. S2F) and *R. sphaeroides* (Supplementary Fig. S2G). The fact that micromolar concentrations of SkQ1 inhibited both Gram-positive (*B. subtilis, Mycobacterium sp*. and *S. aureus)* and Gram-negative (*P. phosphoreum* and *R. sphaeroides)* bacteria supported our assumption that the cell envelope permeability depended on the operation of membrane transporters rather than on structural features of the permeability barrier.

### Effect of SkQ1 may be based on membrane depolarization

**Figure 5:**
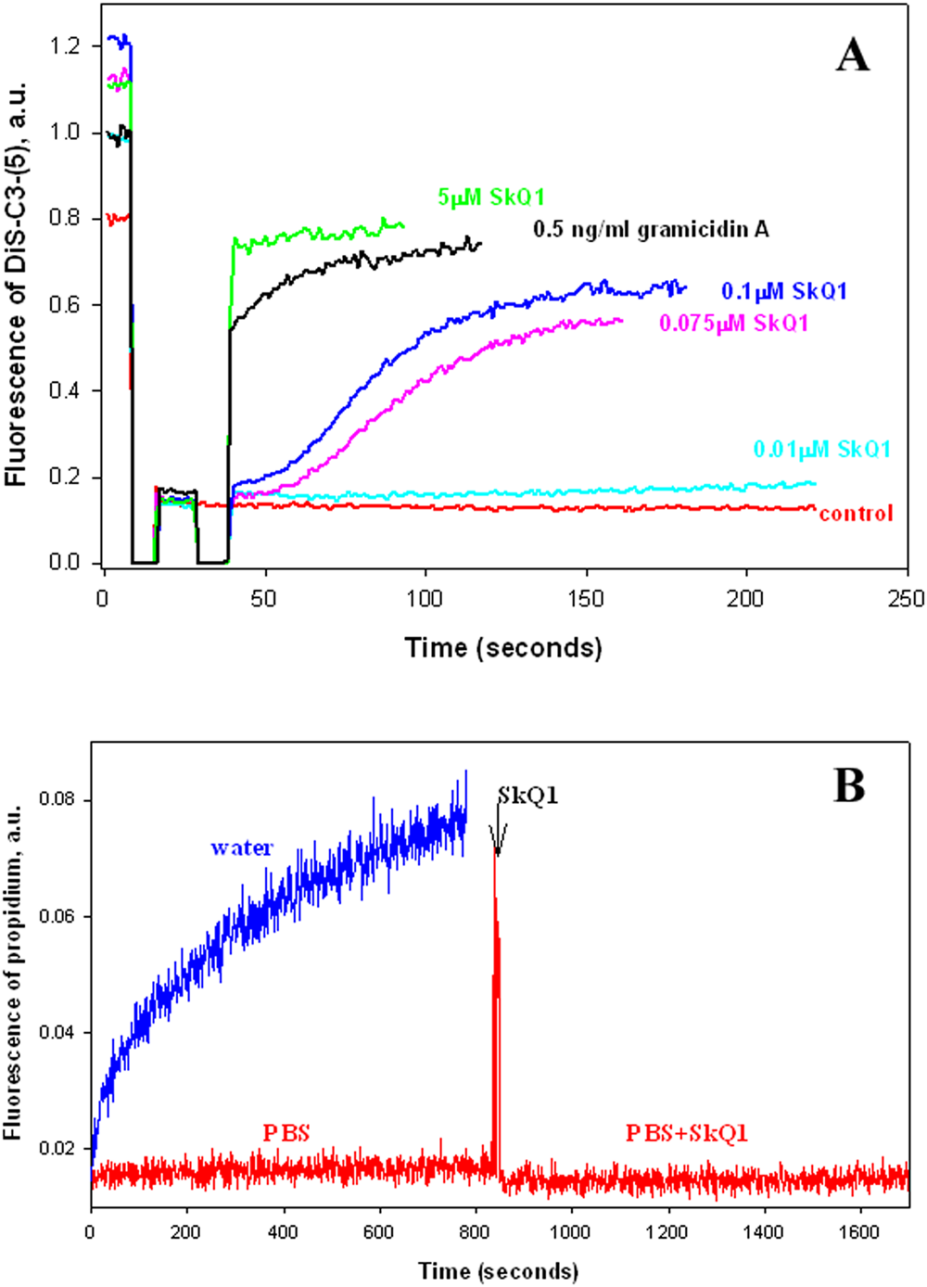
(**A**) Effect of SkQ1 on membrane potential in *B. subtilis*. Changes in the membrane potential were monitored by measuring fluorescence of DiS-C_3_-(5) (10 µM) in PBS buffer. Gramicidin A concentration, 0.5 ng/ml. (**B**) Propidium iodide membrane permeability was tested via its fluorescence at 600 nm at 1 µM. Propidium iodide was present in all samples. Deionized water was used as a positive control of membrane permeabilization. SkQ1 concentration, 1 µM.

Further, we examined the effect of SkQ1 on bacterial membrane potential. In particular, we measured the effect of SkQ1 on the membrane potential of *B. subtilis* which can be estimated from the fluorescence of the potential-sensitive dye DiS-C_3_-(5)^48^. Potential-dependent accumulation of the cationic dye is known to cause its aggregation leading to the fluorescence quenching^49^. As shown in Fig. 5A, submicromolar concentrations of SkQ1 caused a decrease in the membrane potential of *B. subtilis* in the minute time scale (SkQ1 addition marked by an arrow), whereas 5 µM SkQ1 caused a rapid drop of membrane potential to the level observed with the channel-forming antibiotic gramicidin A known to vanish the bacterial membrane potential^50^. Of note, the action of SkQ1 on the membrane potential of *B. subtilis* was not associated with a detergent effect of SkQ1 on bacterial membrane. This conclusion followed from measurements of fluorescence of the cell membrane-impermeant probe propidium iodide, which is known to display a strong increase in fluorescence upon penetration into cells and interaction with cellular DNA^51^. According to Fig. 5B, grey curve, the osmotic shock of *B. subtilis* cells, caused by the addition of the deionized water to the PBS buffer, led to a significant increase in the fluorescence of propidium, whereas no change in the fluorescence intensity occurred in the control (Fig. 5B, red curve). The addition of SkQ1 to *B. subtilis* cells did not provoke any increase in the propidium fluorescence (Fig. 5B, red curve), thereby showing that SkQ1 did not change the cell membrane integrity and propidium could not be expelled by the AcrAB-TolC system.

### SkQ1 does not affect transcription and translation in bacteria

It is well-known that mechanisms of antibiotic-induced bacterial cell death include: (1) inhibition of cell wall synthesis, (2) inhibition of protein synthesis, (3) alteration of cell membranes, (4) inhibition of nucleic acid synthesis, (5) antimetabolite activity, etc.^52^.

**Figure 6:**
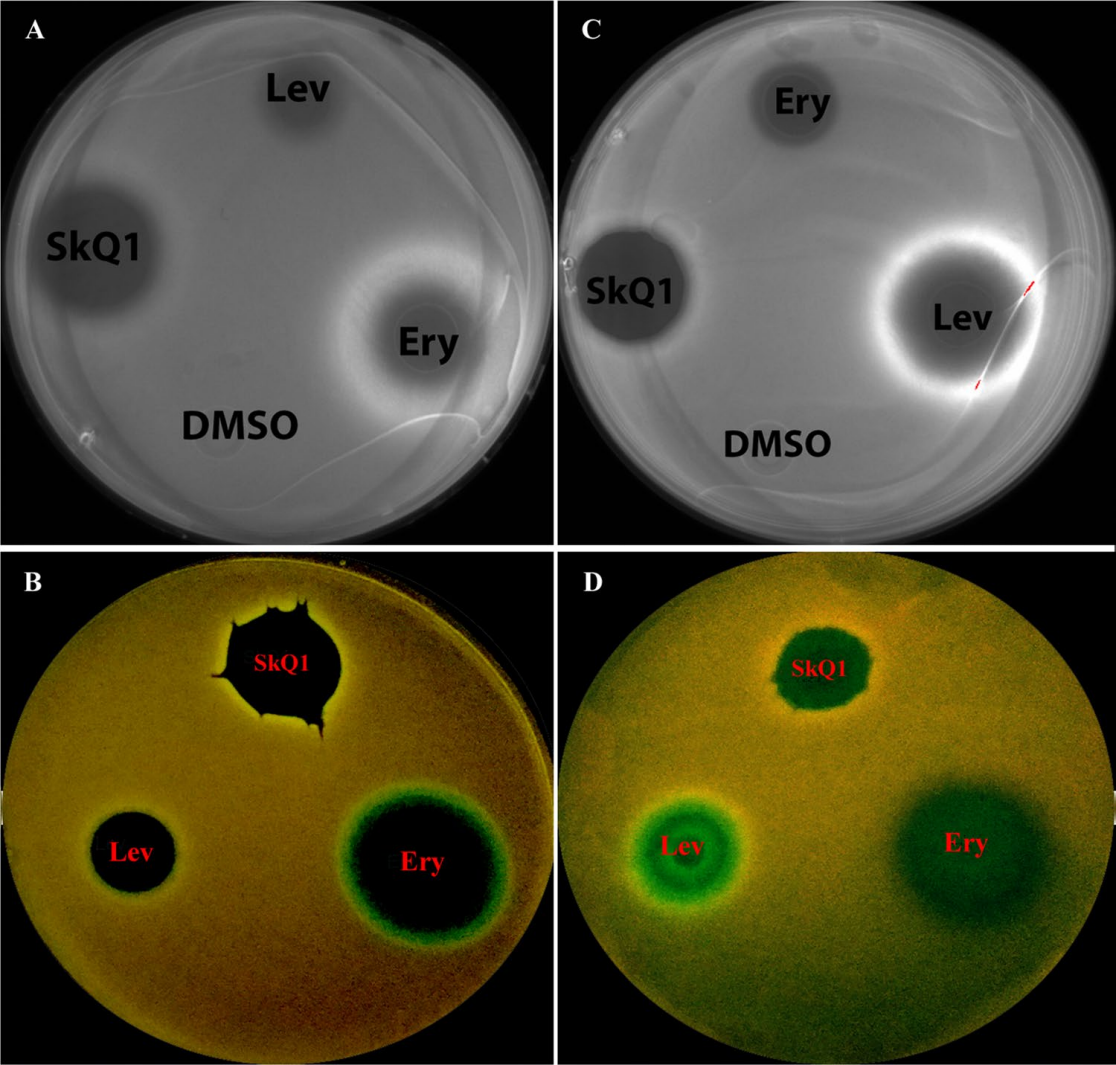
Induction of the translation inhibittion fluorescent reporter (left) or DNA damage fluorescent reporter (right) by SkQ1 on Petri plates. *E. coli ΔtolC* transformed with pRFPCER-TrpL2A (left) or pRFPCER-sulA (right) exhibited red fluorescence owing to RFP expression. SkQ1; erythromycin (ery) and levofloxacin (lev) were spotted on agar. Rings of cerulean fluorescence (cyan-green) were formed under the influence of the antibiotic causing ribosome stalling (left panel), and under the influence of the DNA-damaging antibiotic (right panel). Petri dishes were illuminated at UV (254-nm) and photographed by a digital camera (bottom panels) while the signal of cerulean protein fluorescence was detected in Cy2 channel by means of ChemiDoc (top panel).

To test a possible inhibitory effect of SkQ1 on the translation, the dual reporter system pRFPCER-TrpL2A^53^ was applied to *ΔtolC E. coli*. For this test, we used erythromycin (antibiotic that causes the translation arrest but neither causes a misreading nor affects translation by other mechanisms) and levofloxacin (as a control). After overnight incubation, clear inhibition zones became visible on the bacterial lawn (Fig. 6B, black circle areas), while the bacterial lawn emitted red light due to expression of the red fluorescence protein (RFP). Antibiotics causing ribosome stalling, such as erythromycin, elicited the formation of a bright green ring. The ring was better seen with a Cy2 filter set on panel A of Fig. 6. Antimicrobials, unable to cause ribosome stalling (SkQ1, levofloxacin), did not induce rings of cerulean fluorescence due to lack of the protein expression (Fig. 6A). Therefore, SkQ1 toxicity towards bacterial cells was not associated with inhibition of the translation. An *in vitro* translation test with luciferase mRNA and an extract from *E. coli* cells supplemented with ribosomes showed that SkQ1 was of no effect in agreement with the *in vivo* reporter pRFPCER-TrpL2A observation (data not shown).

Similarly, to test a possible DNA-damaging effect of SkQ1, we used the dual reporter system pRFPCER-sulA. For this test, we selected levofloxacin (DNA gyrase inhibitor, which damages DNA) and erythromycin (as a control). A ring of bright cerulean fluorescence was elicited by the DNA-damaging antibiotic (levofloxacin), but not by erythromycin unable to cause DNA damage (Fig. 6C).

Of note, the above methods allow the detection of *in vivo* transcription and translation defects at sublethal concentrations of antibiotics irrespective of multiple inhibition of many functions including transcription and translation under these conditions^53^. The rings of the reporter’s fluorescence highlight the appearance of transcription or translation defects relying on still functioning of these systems at sublethal doses of antibiotics. In the case of using the reporters for *in vivo* transcription and translation defects at sublethal concentrations of SkQ1, we did not observe reporters’ rings suggesting that SkQ1 did not exhibit direct effect on transcription and translation. Therefore, the mechanism of SkQ1 toxicity towards bacterial cells presumably was not associated with DNA damage or translational defects.

### SkQ1 does not exert significant cytotoxic effect on mammalian cells

**Figure 7:**
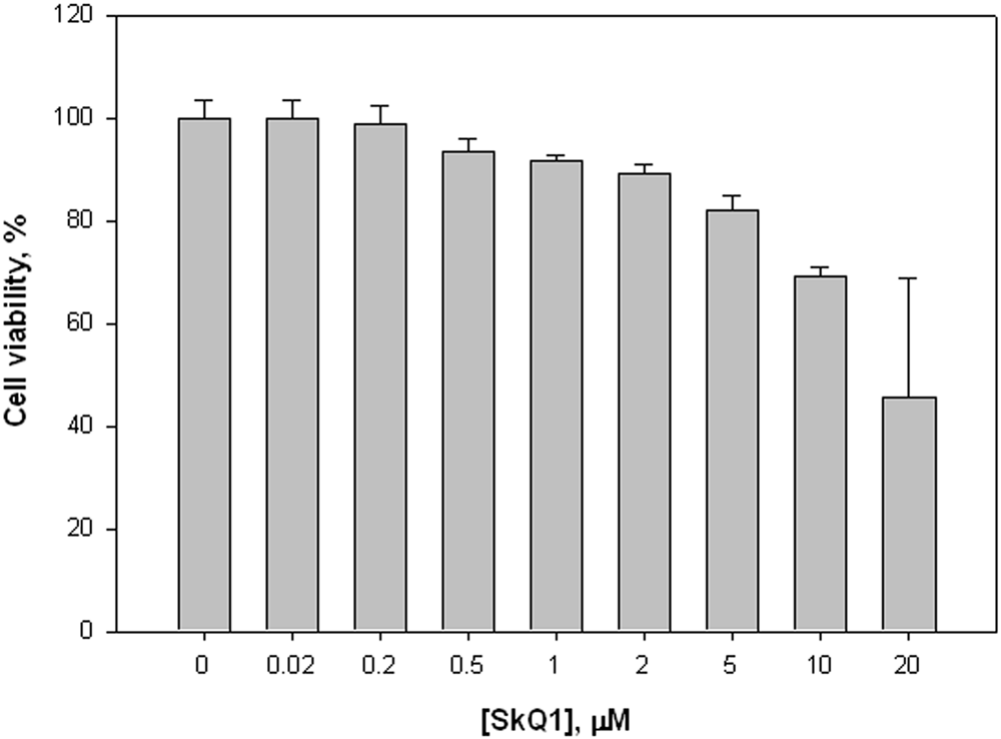
Viability of HeLa cells after the addition of SkQ1. HeLa cells were incubated for 17 h. Cell viability was determined with Cell Titer-Blue reagent (Promega).

In view of the high antibacterial activity of SkQ1 observed in the present study, it was of importance to estimate cytotoxicity of SkQ1 for mammalian cells. For this purpose, we used human immortalized HeLa cells culture. In the 0.01–3 μM concentration range, a significant cytotoxic effect of SkQ1 on HeLa cells was not detected (Fig. 7). Reduction of cell viability did not exceed 10% for 21–24 hours, which supported the idea that SkQ1 could serve as an antibiotic suitable for treating bacterial infections in humans.

## Discussion

SkQ1 demonstrated very efficient antibiotic activity, inhibiting growth of *B. subtilis*, *S. aureus, P. phosphoreum, R. sphaeroides* and *Mycobacterium sp*. at micromolar concentrations. Importantly, SkQ1 acted as a bactericidal agent at concentrations higher than MIC, whereas it acted as a bacteriostatic agent under the conditions when its level was not sufficient to cause the collapse of membrane potential (Fig. 1). Values of the MIC of SkQ1 (Table 1) found here for *B. subtilis* and *S. aureus* were lower than (or comparable to) those of several conventional antibiotics (including vancomycin, azithromycin, chloramphenicol, streptomycin, and kanamycin)^54, 55^. The value of SkQ1 MIC for *Mycobacterium sp*. was also lower than those of the well-known antibiotics azithromycin, levofloxacin, imipenem, trimethoprim, amikacin and many others^56^.

**Table 1:** Susceptibility of bacterial strains to SkQ1: Minimal Inhibitory Concentration (MIC) and Minimal Bactericidal Concentration (MBC) measurements.

	MIC (µg/mL)	MBC (µg/mL)
***B. subtilis***	0.6	N/D
***S. aureus***	0.6	2.4
***Mycobacterium sp*** *.*	0.6	N/D
***E. coli*** WT	19	38
***E. coli*** Δ (AcrD, AcrE, AcrF, MacA, MacB, MdtA, MdtB, MdtC, MdtE, MdtF, EmrA, EmrB)	19	N/D
***E. coli*** ΔTolC	0.6	2.4
***E. coli*** Δ (AcrA, AcrB)	0.6	N/D

The fact that MIC of SkQ1 for various Gram-negative and Gram-positive bacteria, as well as for *E. coli* AcrAB-TolC deletion mutants, does not exceed 2 μM, points to high permeability of SkQ1 through bacterial cell envelopes, irrespective of their sophisticated structural diversity. On the other hand, the presence of certain transporters, such as AcrAB-TolC, in the cell envelope, that counteract the ability of bacteria to electrophoretically accumulate the SkQ1 cations, can drastically lower the antimicrobial effect of SkQ1.

Actually, higher concentrations of SkQ1 were required for the inhibition of *E. coli* growth, which was apparently due to the ability of the AcrAB-TolC system to actively export SkQ1 from *E. coli* cells. Our experiments pointed at the possibility to improve sensitivity of *E. coli* cells to SkQ1 by adding AcrAB-TolC inhibitors or using SkQ1 together with other AcrAB-TolC substrates.

The antibacterial action of SkQ1 and MitoQ is obviously related to the antimicrobial activity of alkyl trimethyl^3^, alkyl trihexyl^5^ and alkyl triphenyl^7, 8^ phosphonium salts, which was attributed, in particular, to membrane-perturbing effects of QPC^6, 9^, similar to QAC^4, 57–59^. Of note, as a pro- or antioxidant, SkQ1 might be involved in ROS-production/suppression. However, similar sensitivity of the *ΔtolC E. coli* mutant to C_12_-TPP and SkQ1 evidenced against this possibility.

**Figure 8:**
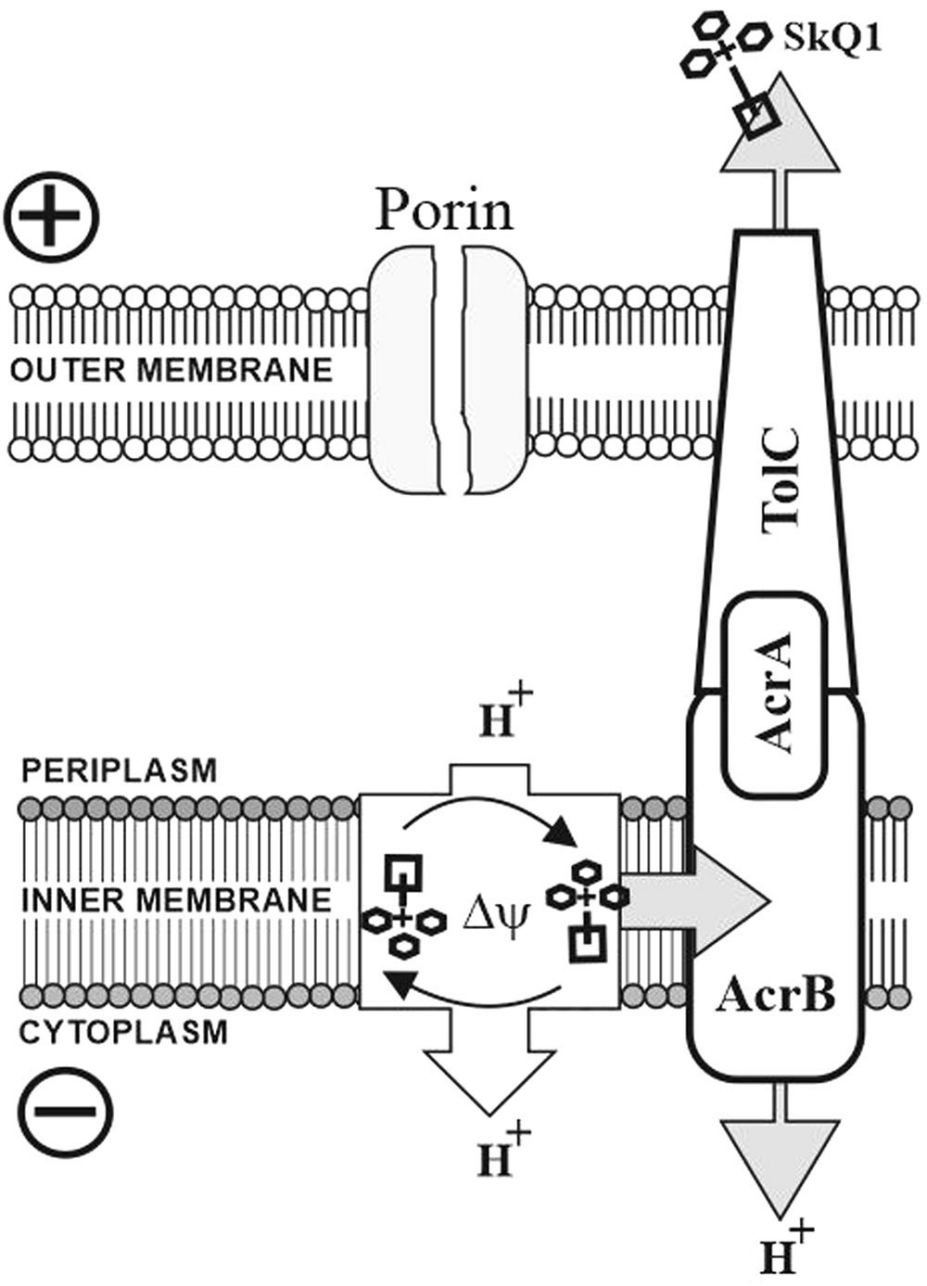
The SkQ1-mediated protonophorous cycling of fatty acids in bacterial membrane. In the absence of SkQ1-expelling transporters, SkQ1 acts as protonophore-like uncoupler with the help of endogenous free fatty acids (left). Export of SkQ1 from the bacterial cell by means of AcrA, AcrB, and TolC pump (right).

The mechanism of the bactericidal action of SkQ1 could be also ascribed to its ability to suppress bacterial bioenergetics by collapsing membrane potential^22, 23^ through activation of protonophorous uncoupling mediated by endogenous fatty acids^32^ (Fig. 8). It is known that protonophores uncouple oxidative phosphorylation by facilitating proton transfer across lipid bilayers. Notably, SkQ1 is not a protonophore, but may perform protonophore-like action in combination with fatty acids^32^, which are abundant in cellular and mitochondrial membranes. According to the hypothesis of the protonophore-like uncoupling in mitochondria with the help of fatty acids^32^, SkQ1 import by bacteria is driven by the electrical potential gradient, negative inside bacterium. Then SkQ1 returns back in the form of the SkQ1 cation/anion pair, thus providing rate-limiting outward transport of the anionic form of the fatty acids. The protonophorous cycle is completed by import of the protonated anion with its deprotonation to anion and H^+^ on the inner surface of the inner bacterial membrane (Fig. 8).

Remarkably, our findings highlight the crucial role of multidrug-resistant pumps’ composition and their ability to expel biocides, but not the passive permeability of the outer cell structures in the overall mechanism of resistance of *E. coli* towards the MTA. According to the current paradigm, the AcrAB-TolC active center is formed by amino-acid residues of all three proteins. Besides, TolC and AcrA also interact with AcrD^60^. Based on our findings that deletion of AcrD did not affect the resistance to SkQ1, and that deletions of AcrA or AcrB dramatically reduced the resistance of the parent strain to SkQ1, we can conclude that the amino-acid residues responsible for interaction with SkQ1 are mainly located on the AcrB part of the tripartite complex.

SkQ1 is a unique artificial substrate that is recognized only by AcrAB-TolC. In contrast to other substrates of AcrAB-TolC, SkQ1 is not expelled by other transporters, which enables one to use SkQ1 in high throughput AcrAB-TolC inhibitors screening. The search for AcrAB-TolC inhibitors is of high importance because application of inhibitors together with antibiotics could prevent expelling the antibiotics out of cell. Such combined therapy would significantly increase effectiveness of antimicrobial therapy and safety for patients.

Noteworthy, SkQ1 contains not only a strong antimicrobial moiety but also an antioxidant moiety targeting mitochondrial reactive oxygen species. It has been recently suggested that SkQ1 can be used in the course of treatment of bacteria-induced pyelonephritis for reducing mitochondrial reactive oxygen species, thereby protecting mitochondrial integrity and lowering kidney damage^61^. Remarkably, some quinone derivatives demonstrated antimicrobial activity towards clinical isolates of Gram-negative and Gram-positive bacteria^62, 63^, therefore SkQ1 can be reasonably considered as a “hybrid” antibiotic.

Based on the present study, it can be suggested that SkQ1 may be effective in protection of the infected mammalian organ by killing invading bacteria on one side, and curing damaged host cells on the other side. Thus, SkQ1 can be considered as a new type of dual-acting “hybrid” antibiotic. The absence of cytotoxicity of 1–2 µM SkQ1 for human and animal cells means that SkQ1 might be safely used to treat many bacterial infections.

## Materials and Methods

SkQ1, MitoQ and C_12_TPP were synthesized, as described in ref. 64. Ethidium bromide and some components of bacterial media (peptone, agar and yeast extract) were purchased from Heliсon Company (Moscow, Russia), and Mueller-Hinton medium was purchased from HiMedia Laboratories (Mumbai, India). Other reagents were from Sigma-Aldrich (USA).

### Bacterial strains

Standard laboratory strains *Bacillus subtilis* subs. *subtilis* Cohn 1872, stain BR151 (trpC2 lys-3 metB10), and *Escherichia coli* Castellani and Chalmers 1919, strain W3110 (F- lambda-IN(rrnD-rrE)1 rph-1) were used.

Deletion strains ECK3026 (devoid of the *tolC* gene), ECK0457 (devoid of the *acrA* gene), ECK0456 (devoid of the *acrB* gene), ECK2465 (devoid of the *acrD* gene), ECK3252 (devoid of the *acrE* gene), ECK3253 (devoid of the *acrF* gene), ECK2070 (devoid of the *mdtA* gene), ECK2071 (devoid of the *mdtB* gene), ECK2072 (devoid of the *mdtC* gene), ECK3497 (devoid of the *mdtE* gene), ECK3498 (devoid of the *mdtF* gene), ECK0869 (devoid of the *macA* gene), ECK0870 (devoid of the *macB* gene), ECK2679 (devoid of the *emrA* gene) and ECK2680 (devoid of the *emrB* gene) were kindly provided by Dr. H. Niki, National Institute of Genetics, Japan^65^.

*Photobacterium phosphoreum* was kindly provided by Dr. A.D. Ismailov, Moscow State University. *Rhodobacter sphaeroides* (van Niel 1941), Imhoff *et al*., 1984 (DSM158) was kindly provided by Prof. Dr. G. Klug, Institut fuer Mikrobiologie und Molekularbiologie Justus Liebig Universitaet, Giessen, Germany. *Staphyllococcus aureus* Rosenbach 1884 (entry #144) and *Mycobacterium sp*. (entry #377) were obtained from the Microorganisms Collection of the Moscow State University.

Bacterial cells were grown at 30 °С or 37 °С in LB^66^ medium at 140 rpm shaking frequency. *Rhodobacter sphaeroides* cells were grown in Sistrom’s medium A^67^, *Photobacterium phosphoreum* cells were grown in a LB-SWR medium (LB medium with 20 g/l sea salt).

### Growth suppression assay

Overnight bacterial cells cultures were diluted in fresh LB media. 200 µl of bacterial cell cultures (5 * 10^5^ cells/ml) were inoculated into 96-well plates (Eppendorf AG, Hamburg, Germany) and SkQ1, MitoQ or Cn-TPP were added at concentrations of 0.5–200 μM. Cells were left to grow for 21 hours at 37 °C. Optical densities at 620 nm were obtained by using a Thermo Scientific Multiskan FC plate reader with an incubator (Thermo Fisher Scientific, USA). All experiments were performed at least in triplicates.

### MIC and minimal bactericidal concentration (MBC) determination

MICs for SkQ1 were determined by Mueller-Hinton broth microdilution, as recommended by CLSI in Methods for Dilution Antimicrobial Susceptibility Tests for Bacteria that Grow Aerobically, Approved Standard, 9th ed., CLSI document M07-A9, using in-house-prepared panels. The compounds were diluted in a 96-well microtiter plate to final concentrations ranging from 0.5 to 64 µM in 250-ml aliquot of the bacterial suspension (5 × 10^5^ CFU/ml) followed by the incubation at 37 °C for 18 h. MIC was determined as the lowest concentration that completely inhibited the bacterial growth. Bacterial growth was observed visually alongside with CFU and OD measurements. Experiments were carried out in triplicate.

MBC was determined as the lowest concentration at which no colonies were found during CFU measurement.

### SkQ1-dependent bacterial growth suppression screening of TolC-requiring transporters

*E. coli* deletion mutants’ panel was selected in accordance with data protein-protein interactions between TolC and other proteins in *E. coli* strains W3110 and MG1655 by using database STRING v.10. Selected bacterial strains belonging to the panel were diluted in fresh LB media after overnight growing. 200 µl of bacterial cell cultures (5 * 10^5^ cells/ml) were inoculated into 96-well plates. Preselected SkQ1 concentrations (5 µM, 30 µM and 50 µM) were added to each mutant and optical density at 620 nm was measured using a Thermo Scientific Multiskan FC plate reader. Cells were left to grow for 21 hours at 37 °C and optical density at 620 nm was measured. All experiments were performed at least in triplicates.

### Accumulation of ethidium bromide

Bacterial cells were prepared as described^41^ and resuspended in PBS. For measuring cell membrane leakage, bacterial cells were resuspended in deionized water (Milli-Q water purification system, EMD Millipore, Billerica, MA). Ethidium bromide was added (20 μg/ml) and the change of fluorescence intensity was recorded at 600 nm on Fluorat-02-Panorama fluorimeter (Lumex Instruments, Russia) upon excitation at 530 nm. SkQ1 was added to reach the concentration of 50 μM.

### Propidium iodide membrane permeability test

Bacterial cells were prepared as described^41^ and resuspended in PBS. For measuring cell membrane leakage, bacterial cells were resuspended in deionized water. Propidium iodide was added (20 μg/ml) and the change of fluorescence intensity was recorded at 600 nm on Fluorat-02-Panorama fluorimeter (Lumex Instruments, Russia) upon excitation at 530 nm. SkQ1 was added at 20 μM concentration.

### Measurement of *B. subtilis* membrane potential

Membrane potential in *B. subtilis* was estimated by measuring fluorescence of the potential-dependent probe DiS-C3-(5)^33^. *B. subtilis* from the overnight culture were seeded into fresh LB medium, followed by growth for 24 h until reaching the optical density 0.8 at 600 nm. Then the bacteria were diluted 20-fold in a buffer containing 100 mM KCl, 10 mM Tris, pH 7.4. Fluorescence was measured at 690 nm (excitation at 622 nm) by using a Fluorat-02-Panorama fluorimeter.

### Fluorescence correlation spectroscopy

Fluorescence correlation spectroscopy (FCS) measurements were carried out with a homemade FCS setup^43^ including an Olympus IMT-2 inverted microscope with a 40x, NA 1.2 water immersion objective (Carl Zeiss, Jena, Germany). A Nd:YAG solid state laser was used for excitation of SRB at 532 nm. The fluorescence that passed through an appropriate dichroic beam splitter and a long-pass filter was imaged onto a 50-μm core fiber coupled to an avalanche photodiode (PerkinElmer Optoelectronics, Fremont, CA). The signal from an output was correlated by a correlator card (Correlator.com, Bridgewater, NJ). The data acquisition time was 30 s. The experimental data were obtained under stirring conditions which increased the number of events by about three orders of magnitude thus substantially enhancing the resolution of the method. Peak intensities of fluorescence traces with the sampling time of 25 μs were analyzed using WinEDR Strathclyde Electrophysiology Software designed by J. Dempster (University of Strathclyde, UK). The software, originally designed for the single-channel analysis of electrophysiological data, enables one to count the number of peaks [n(F > F_0_)] of the FCS signal having amplitudes higher than the defined value F_0_. A program of our own design with a similar algorithm (coined Saligat; provided on request) was also used.

### DNA damage and inhibition of translation tests

The experiment was carried out as described^53^. The overnight culture of Δ*tolC E. coli* cells, transformed by pRFPCER-TrpL2A (translation reporter) or pRFPCER-sulA (DNA damage reporter), was diluted to 0.05 to 0.1 OD (590 nm) units with fresh LB medium supplied with ampicillin 100 μg/ml and plated on LB agar medium (ampicillin 100 μg/ml). After drying the plate, 1 μl of antibiotic or a disk soaked in antibiotic solution was added at the following concentrations: erythromycin at 10 mg/ml, levofloxacin at 3 µg/ml and SkQ1 as a 10 mM solution. After overnight incubation at 37 °C, rings of inhibition zones formed by cerulean fluorescence protein became visible as green fluorescence at subinhibitory concentrations of antibiotics causing ribosome stalling or the DNA damage. Far from inhibition zones, the bacterial lawn showed red fluorescence due to higher expression of the red fluorescence protein over the cerulean fluorescence protein.

As a negative control of the translation inhibition, we used levofloxacin as DNA gyrase inhibitor; as a negative control of the DNA damage we used erythromycin as a translation inhibitor.

The signal of fluorescence from the Petri dishes was visualized using UV-lamp illumination UV (254-nm), photographed by a digital camera and the signal from cerulean protein was detected in Cy2 channel by means of ChemiDoc.

### *In vitro* translation test

*In vitro* translation of luciferase mRNA was carried out in S30 extract from *E. coli* cells supplemented with ribosomes, according to ref. 68 with minor modifications. *E. coli* S30 extract was prepared according to ref. 69 procedure with minor modifications. Luciferase mRNA was presynthesized *in vitro* in accordance with ref. 68.

Activity of the synthesized luciferase was detected at time points of 15 min. SkQ1 at a concentration of 10 μM was added to translation medium, erythromycin at a concentration of 10 μg/ml served as a control of the translation inhibition.

### Experiments with HeLa cell line

HeLa cells were cultured in Dulbecco’s modified Eagle’s medium (DMEM) supplemented with 10% fetal calf serum, streptomycin (100 U/ml), and penicillin (100 U/ml). Viability of HeLa cells was assayed using CellTiter-Blue Reagent (Promega). Cells were prepared at 100 µl/well in a 96-well plate and cultured for 24 hours at 37 °C. Cells were treated with SkQ1 for 17 hours after that Cell Titer-Blue (20 µl/well) was added and cells were incubated for 1 hour before recording fluorescence (544(20) Ex/590(10)Em) using a Fluoroskan Ascent™ Microplate Fluorometer (Thermo).

### Bioethics

All experimental procedures were reviewed and approved by the Institutional Ethics Committee of the A. N. Belozersky Institute.

### Math, bioinformatics and statistical analysis

For bioinformatic study we applied *STRING* v.10 database located at European Molecular Biology Laboratory (EMBL, http://string.embl.de/), The data were expressed as mean ± SD.

## Electronic supplementary material

Mitochondria-targeted antioxidants as highly effective antibiotics
