# Supplementary material for: Mitochondria-targeted antioxidants as highly effective antibiotics
Source: Sci Rep. 2017 May 3;7:1394. doi: 10.1038/s41598-017-00802-8 (PMC5431119; doi:10.1038/s41598-017-00802-8)

Supplementary Information

Mitochondria-targeted antioxidants as highly effective antibiotics

Pavel A. Nazarov[1*], Ilya A. Osterman[2, 3], Artem V. Tokarchuk[1], Marina V. Karakozova[4], Galina A. Korshunova[1], Konstantin G. Lyamzaev[1], Maxim V. Skulachev[1, 5], Elena A. Kotova[1], Vladimir P. Skulachev[1, 5], Yuri N. Antonenko[1]

1. Belozersky Institute of Physico-Chemical Biology, Lomonosov Moscow State University, Moscow, 119991, Russia.
2. Department of Chemistry, Lomonosov Moscow State University, Moscow, 119991, Russia.
3. Skolkovo Institute of Science and Technology, Skolkovo, 143026, Russia.
4. Vavilov Institute of General Genetics, Russian Academy of Science, Moscow, 117971, Russia.
5. Institute of Mitoengineering, Lomonosov Moscow State University, Moscow, 119991, Russia.

* nazarovpa@gmail.com

Table of Contents:

1. Chemical structures of cationic SkQ1 and MitoQ ……………………………… S2
2. Effect of 1 μM SkQ1 and 1 μM MitoQ on the growth of bacteria ………………S3
   A. Effect of 1 μM SkQ1 and 1 μM MitoQ on the growth of *B. subtilis*…………S3
   B. Effect of SkQ1 on the growth of *Mycobacterium sp*…………………………S4
   C. Effect of SkQ1 on the growth of *S. aureus*…………………………………...S5
   D. Effect of SkQ1 on the growth of *P. phosphoreum*…………………………...S6
   E. Effect of SkQ1 on the growth of *R. sphaeroides*……………………………..S7
   F. Effect of 1-50 μM SkQ1 on the growth of WT *E. coli*……………………….S8
   G. Effect of 1 μM SkQ1 on the growth of *E. coli* WT and Δ*tolC*………………S9
3. Growth of *ΔtolC E. coli* in different media………………………………………S10

Supplementary Figure S1.

Chemical structures of cationic SkQ1 and MitoQ.

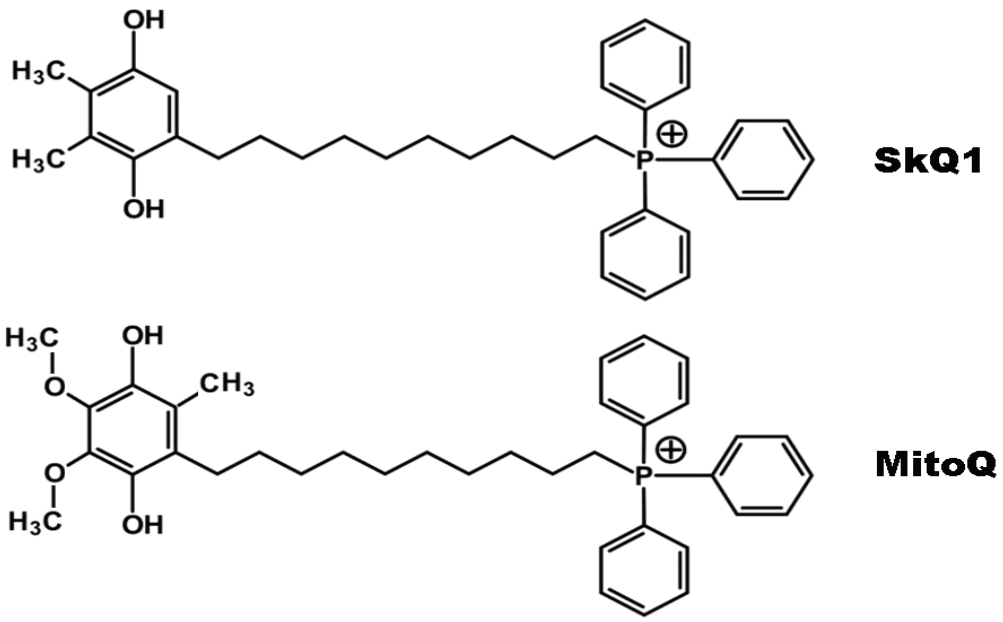

Supplementary Figure S2.

Effect of 1 µM SkQ1 and 1 µM MitoQ on the growth of bacteria.

Growth effects in LB medium were evaluated by hourly measured absorbance at 620 nm during incubation at 37^0C by a Multiskan FC 96-plate reader. Open triangles and circles show control growth of the bacteria without inhibitors which reached the OD_{620} values of 0.5-1 depending on the microbes. The data points represent mean ±SD of three experiments.

Figure S2A

Effect of 1 µM SkQ1 and 1 µM MitoQ on the growth of *B. subtilis*.

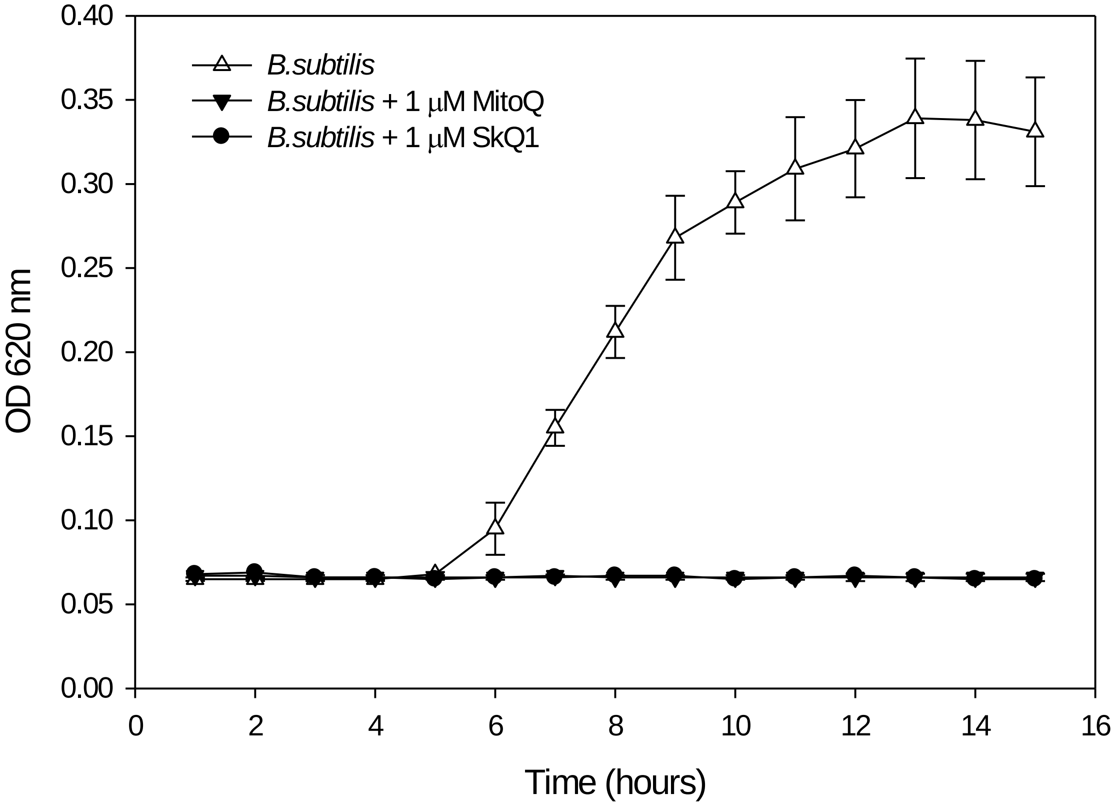

Figure S2B

Effect of 1-50 μM SkQ1 on the growth of WT *E. coli*.

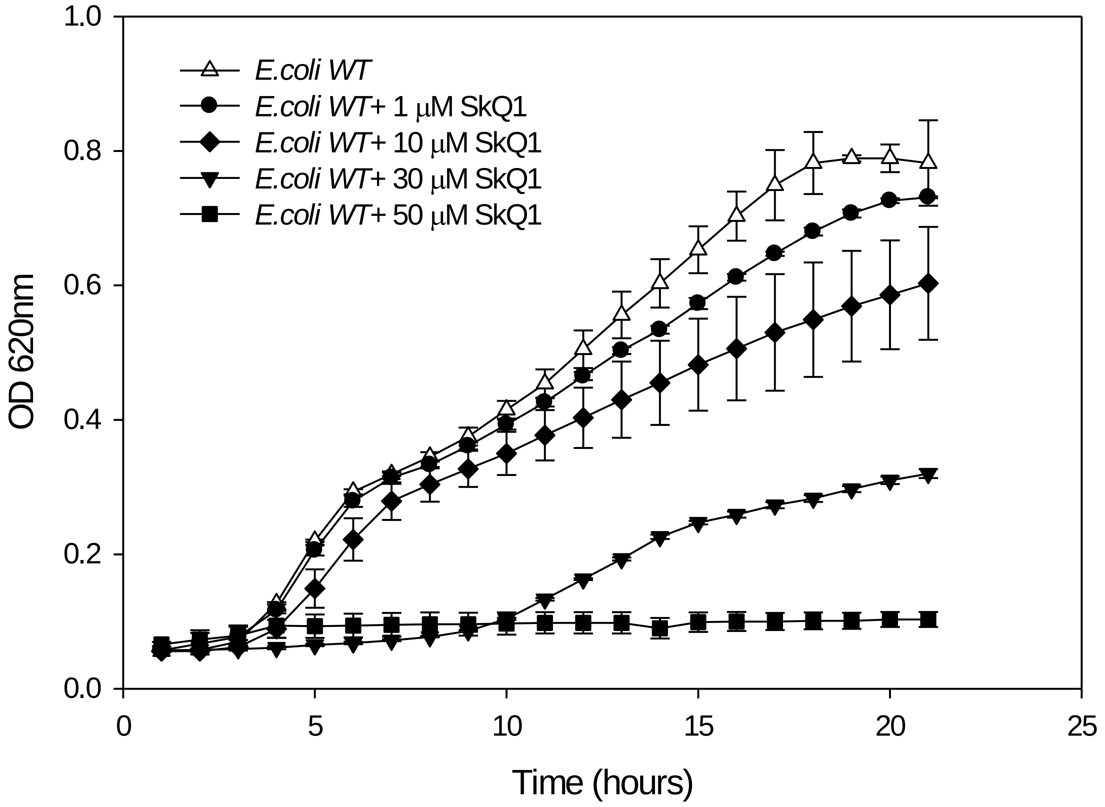

Figure S2C

Effect of 1 μM SkQ1 on the growth of *E. coli* WT and Δ*tolC*.

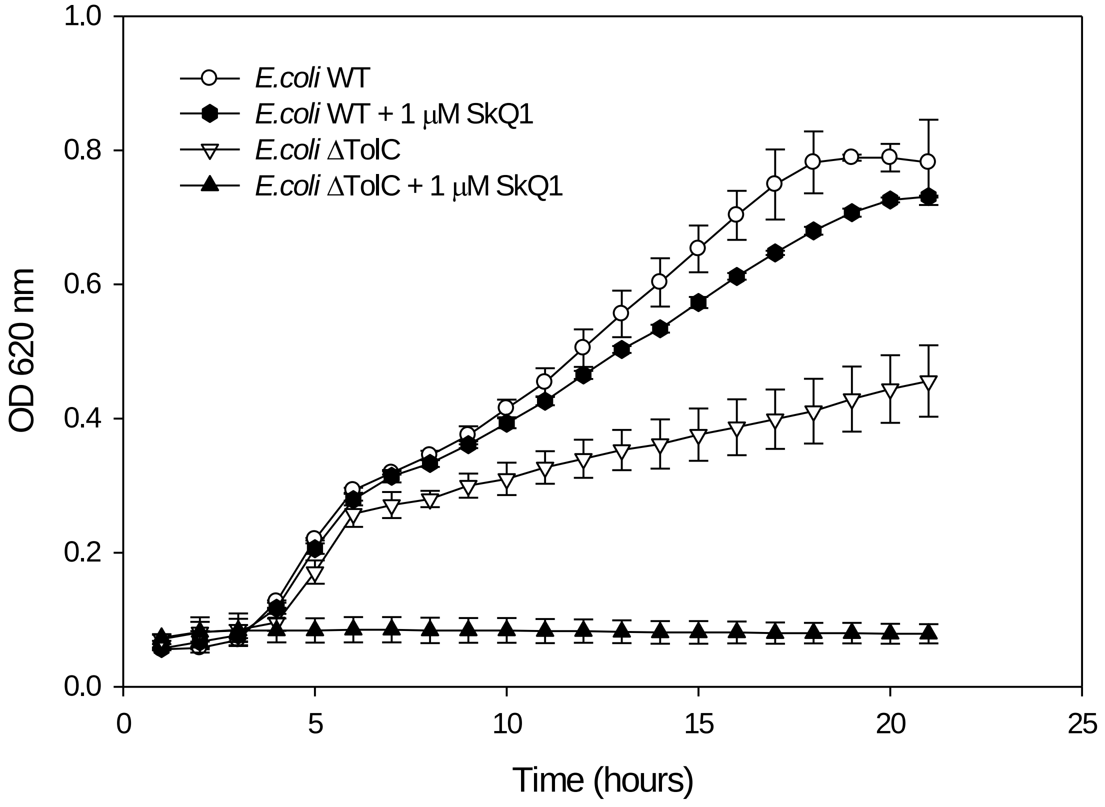

Figure S2D

Effect of SkQ1 on the growth of *Mycobacterium sp*.

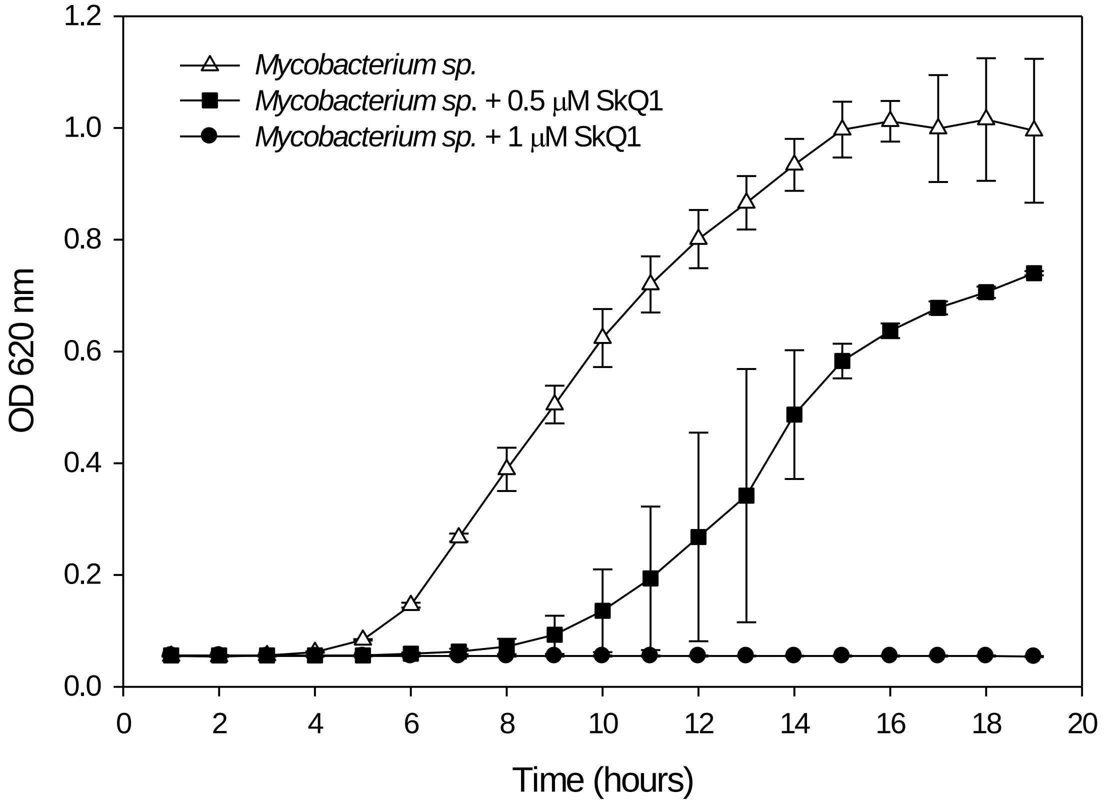

Figure S2E

Effect of SkQ1 on the growth of *S. aureus*.

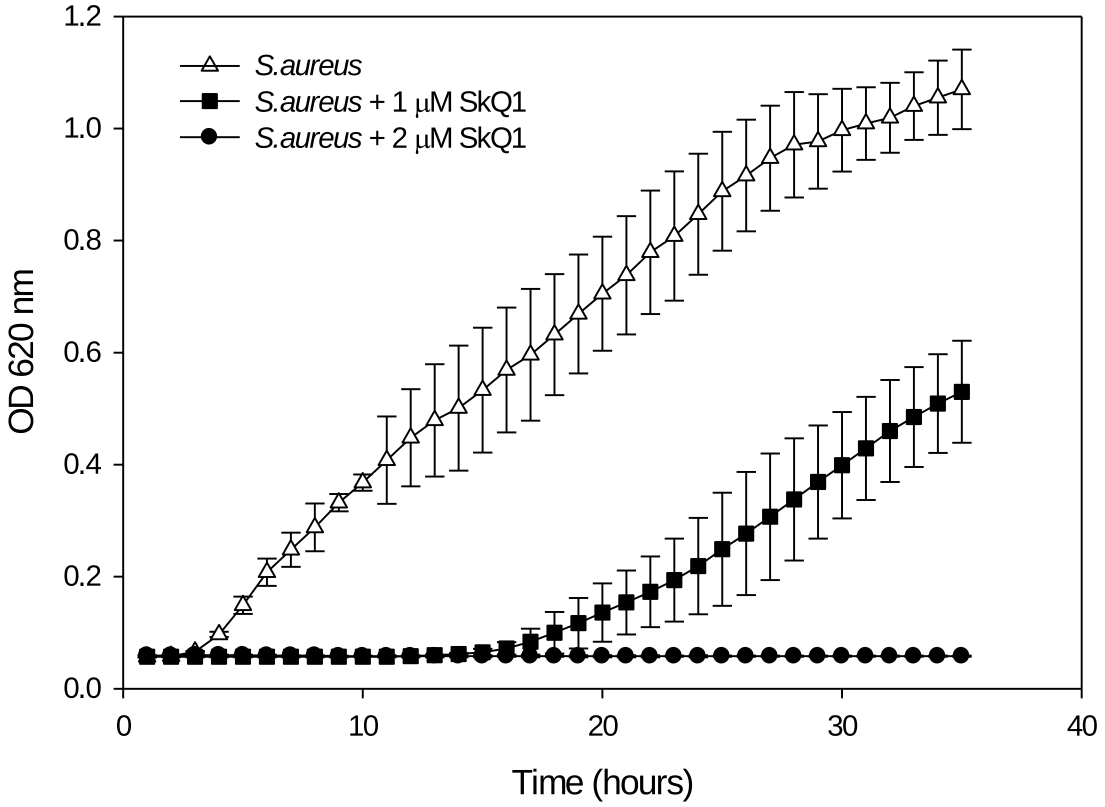

Figure S2F

Effect of SkQ1 on the growth of *P. phosphoreum*.

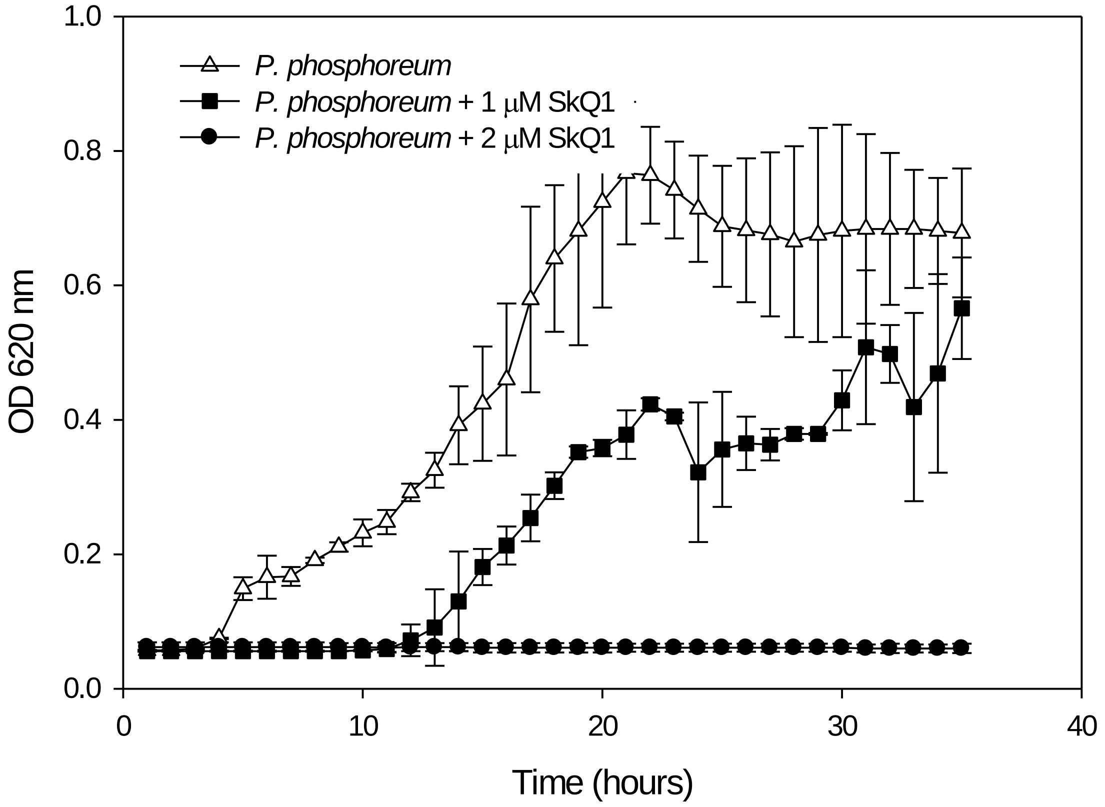

Figure S2G

Effect of SkQ1 on the growth of *R. sphaeroides*.

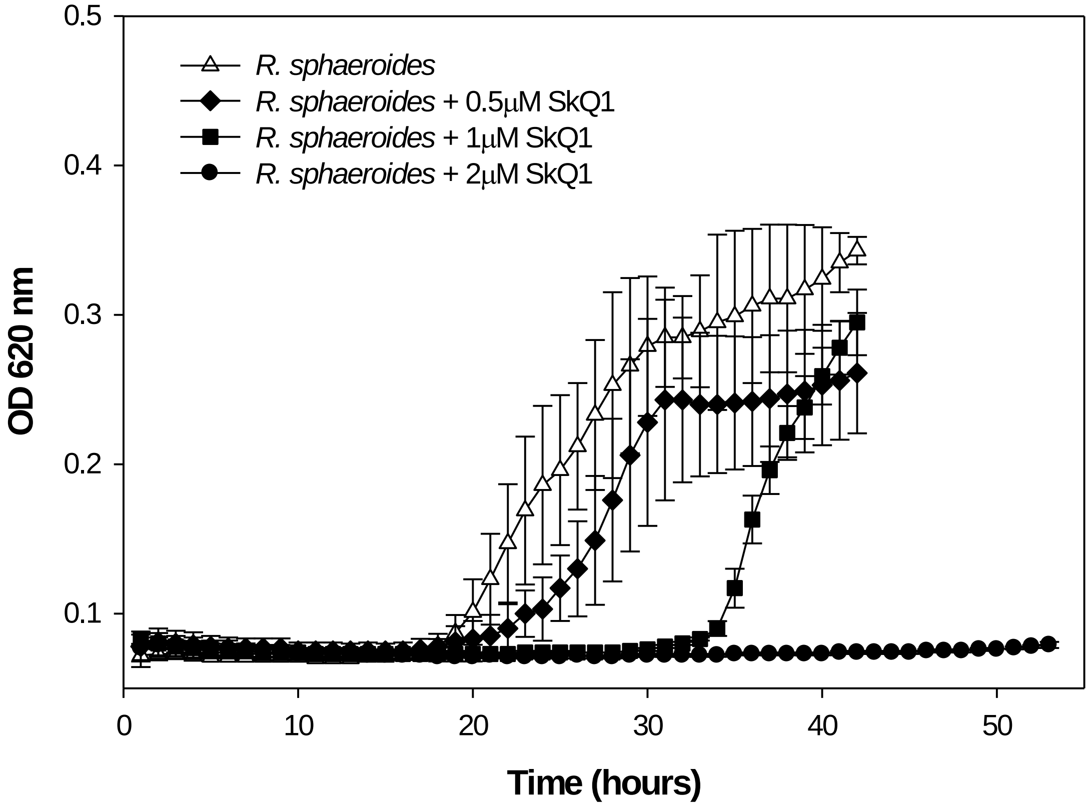

Supplementary Figure S3.

Growth of *ΔtolC E. coli* in different media.

Comparison of growth effects in LB and Mueller-Hinton (MH) media, and Dulbecco's modified Eagle's medium (DMEM) supplemented with 10% fetal calf serum were evaluated by hourly measured absorbance at 620 nm during incubation at 37^0C by a Multiskan FC 96-plate reader. Open triangles, diamonds and circles show control growth of the bacteria without inhibitors. The data points represent mean ±SD of three experiments.

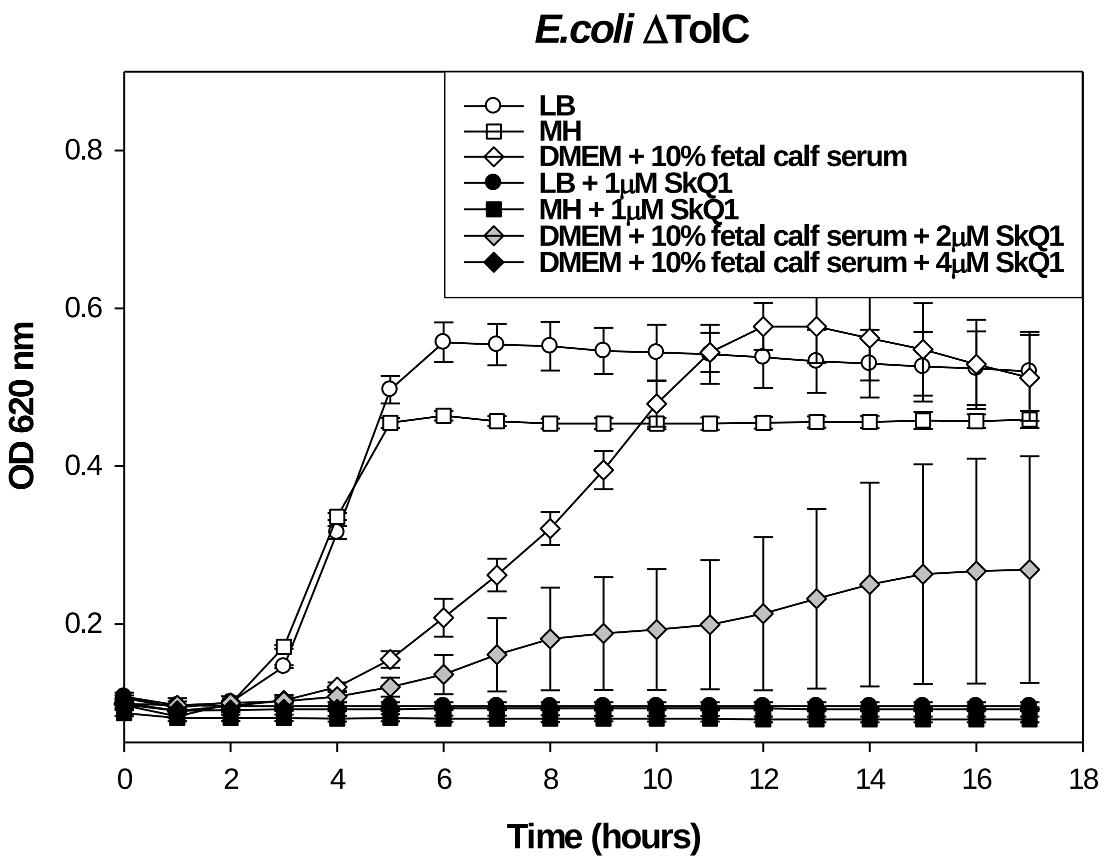

Supplement: Supplementary file 1 — Mitochondria-targeted antioxidants as highly effective antibiotics [file 41598_2017_802_MOESM1_ESM.pdf]
